# Machine Learning Identifies Robust Matrisome Markers and Regulatory Mechanisms in Cancer

**DOI:** 10.3390/ijms21228837

**Published:** 2020-11-22

**Authors:** Anni Kääriäinen, Vilma Pesola, Annalena Dittmann, Juho Kontio, Jarkko Koivunen, Taina Pihlajaniemi, Valerio Izzi

**Affiliations:** 1 Faculty of Biochemistry and Molecular Medicine, University of Oulu, P.O. BOX 8000, FI-90014 Oulu, Finland; anni.kaariainen@oulu.fi (A.K.); vilma.pesola@oulu.fi (V.P.); annalena.dittmann@oulu.fi (A.D.); juho.kontio@oulu.fi (J.K.); jarkko.koivunen@oulu.fi (J.K.); taina.pihlajaniemi@oulu.fi (T.P.); 2 Faculty of Medicine, University of Oulu, P.O. BOX 8000, FI-90014 Oulu, Finland; 3 Finnish Cancer Institute, 00130 Helsinki, Finland

**Keywords:** extracellular matrix, matrisome, cancer, regulatory networks, bioinformatics, big data

## Abstract

The expression and regulation of matrisome genes—the ensemble of extracellular matrix, ECM, ECM-associated proteins and regulators as well as cytokines, chemokines and growth factors—is of paramount importance for many biological processes and signals within the tumor microenvironment. The availability of large and diverse multi-omics data enables mapping and understanding of the regulatory circuitry governing the tumor matrisome to an unprecedented level, though such a volume of information requires robust approaches to data analysis and integration. In this study, we show that combining Pan-Cancer expression data from The Cancer Genome Atlas (TCGA) with genomics, epigenomics and microenvironmental features from TCGA and other sources enables the identification of “landmark” matrisome genes and machine learning-based reconstruction of their regulatory networks in 74 clinical and molecular subtypes of human cancers and approx. 6700 patients. These results, enriched for prognostic genes and cross-validated markers at the protein level, unravel the role of genetic and epigenetic programs in governing the tumor matrisome and allow the prioritization of tumor-specific matrisome genes (and their regulators) for the development of novel therapeutic approaches.

## 1. Introduction

The microenvironment plays a crucial role in all the steps of cancer development, progression and dissemination [1,2,3,4], and the wealth of multi-dimensional, multi-omics data provided by large international consortia such as The Cancer Genome Atlas (TCGA) have drastically changed our understanding of oncogenic processes [5]. These data have naturally led to significant discoveries in the field of tumor microenvironment (TME) too [6,7,8,9,10,11], though only a few have investigated the matrisome [12,13,14,15], which makes up for the non-cellular portion of the TME, at a system level.

The matrisome is an ensemble of extracellular matrix moieties (ECM), together with ECM-associated proteins, ECM regulators, cytokines, chemokines and growth factors, first defined by Naba et al. [16]. As a whole, the matrisome has a paramount importance in cancer, as all the 10 hallmarks of cancers proposed by Weinberg and Hanahan [17] are under the direct control of the matrisome [18]. Identifying and classifying the composition of different tumors’ matrisome is, thus, fundamental to fully understanding the many aspects of tumorigenesis. Unravelling the regulatory mechanisms that govern the tumor matrisome is imperative for the development of novel therapeutic options.

Here we present a bioinformatics pipeline able to handle the tasks of identifying robust matrisome tumor markers (“landmarks”) and their regulators at an unprecedented depth, leveraging on the integration of clinical, phenotypical and molecular data from different sources. With this, we tested the expression of each of the 1027 matrisome genes for association with 24 different tumor types (further subdivided into 96 clinical and molecular phenotypes) and against a huge compendium of mutations, copy number alterations (CNA), transcription factors, gene programs, epigenetic statuses and stromal and immune abundance to infer regulatory mechanisms.

These results identify 210 statistically robust matrisome markers for 74 tumor subtypes, each integrated with one or more regulatory mechanisms. Among the identified matrisome targets, 32 are also of prognostic value and mapping their regulatory mechanisms opens the way to the development of novel targeted therapeutic approaches.

## 2. Results

Our analysis integrates a large amount of data from The Cancer Genome Atlas (TCGA) [19], the miRTar database [20] and several publications and repositories containing information on tumor purity [21], genetic programs’ activity [22] and transcription factors-target interactions [23,24], stacking 8 information “layers” (gene expression, copy number alterations—CNAs, mutations, micro-RNAs (miRNAs), tumor purity, transcription factors, gene programs and methylation) to enable the discovery of matrisome markers and regulators in 24 tumor types, further classified into 74 clinical and molecular subtypes [25].

**Figure 1:**
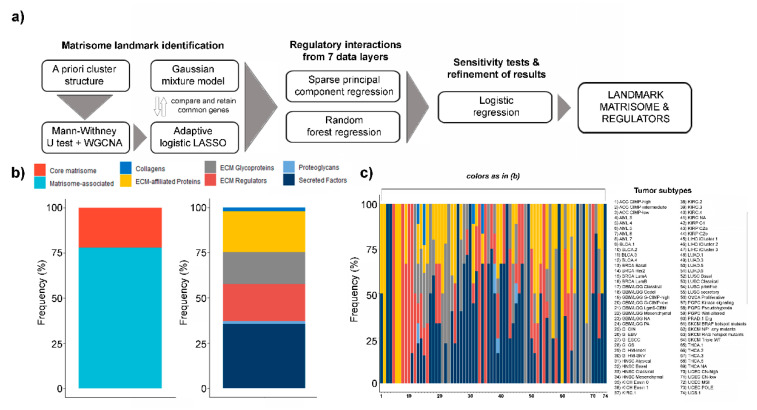
Landmark matrisome gene and regulators. (**a**) Pipeline for the identification of tumor subtype-specific (“landmark”) matrisome genes and their regulators; (**b**) % abundance of core and matrisome associated as well as collagens, extracellular matrix moieties (ECM)-affiliated proteins, ECM glycoproteins, ECM regulators, proteoglycans and secreted factors in landmark genes; (**c**) % abundance of interactions impinging on collagens, ECM-affiliated proteins, ECM glycoproteins, ECM regulators, proteoglycans and secreted factors, by tumor type.

In brief (see Section 4 and Figure 1a), we start from observing that matrisome gene expression implicitly clusters the samples into four large groups: acute myeloid leukemia (LAML, the only hematological cancer in this study), liver hepatocellular carcinoma (LIHC), neuroendocrine tumors, squamous tumors and adenomatous/sarcomatous (all other tumors, Figure S1). Starting from this structure, we mine “landmark” matrisome genes at: (I) the cluster level, combining statistical inference (Mann–Whitney test) and information processing (weighted gene coexpression analysis, WGCNA) [26], and pruning the results with adaptive LASSO regression, and, (II) the tumor subtype level, fitting each matrisome gene into a Gaussian Mixture Model (GMM) and comparing its expression in the tumor subtype vs. the rest of the cohort. The genes that characterize both a tumor subtype and its cluster of origin are then selected and sparse principal component regression (sPCR) and random forest regression (RFR) are independently applied to identify gene regulators among the available information layers. The results are finally tested for robustness in a logistic regression setup applied to each triplet of tumor subtype-matrisome gene-explanatory interaction.

At completion, this pipeline identifies 210 “landmark” matrisome genes (out of 1027, 20.44% of total) whose expression and regulation by 153 diverse regressors (out of 109,863 potential regulatory elements, 0.14% of total) can be robustly imputed to one or more of 74 specific tumor subtypes, for a total of 531 regulatory interactions (Table S1). 

In 52 of the tumor subtypes tested (52/74, 70.3% of total), the landmark genes identified are much more frequently in the top 1st quarter of matrisome gene expression than the other (non-selected) matrisome genes (Figure S2), suggesting that our approach selects for the most highly-expressed matrisome genes in each tumor subtype. The large majority of all selected genes appear only once (155/210, 73.8% of total) or twice (42/210, 20% of total) in the list, confirming the high specificity of our pipeline at identifying unique markers at the tumor-subtype level as well as at the more general cluster level (Table S1 and Figure S3). 45 (45/210, 21.4% of total) of the selected landmark genes are core matrisome while the rest (165/210, 78.6% of total) are matrisome-associated (Figure 1b); in more detail, 6 (6/210, 2.85% of total) are collagens (*COL9A3*, *COL16A1*, *COL5A3*, *COL8A2, COL19A1* and *COL6A1*), 2 (2/210, 0.95% of total) are proteoglycans (*PRG2* and *ACAN*) and the rest are ECM-affiliated proteins (46/210, 21.9% of total), ECM glycoproteins (37/210, 17.6% of total), ECM regulators (42/210, 20% of total) and secreted factors (77/210, 36.7%) (Figure 1b), corroborating our previous report [13] that the structural (core) elements of the matrisome are less frequently specific tumor markers.

Cross-validating these landmark genes at the protein level is unfeasible in matching TCGA data, as the protocol deployed for reverse phase protein array removes insoluble proteins and, thus, removes ECM proteins almost entirely [27]. As an alternative, we resorted to The Human Protein Atlas (THPA) [28], that provides staining profiles for proteins in different human tumor tissues based on immunohistochemistry using tissue microarrays. Even though translating the exact tumor subtypes from TCGA into the much more general tumor categories of THPA is problematic, we could cross-validate 135 genes (135/210, 64.3%). Furthermore, evaluating each landmark gene by the tumor type(s) it belongs to, we obtained non-zero protein signal from 127 out of 194 combinations (65.5% of total) and an average of 40% samples in each tumor type stained positive for any landmark gene, with an average 23% samples in each tumor type staining at either “high” or “medium” levels, thus confirming the general validity and robustness of our approach (Table S2).

At the regulatory interactions’ level, 117 (117/531, 22% of total) impinge on core matrisome [16] while the rest (414/531, 78% of total) on matrisome-associated genes (Figure S4). Considering the major matrisome categories [16], these numbers translate into 6 regulatory interactions (6/531, 1.13% of total) for collagens, 7 for proteoglycans (7/531, 1.31% of total), 104 for ECM glycoproteins (104/531, 19.6% of total), 120 for ECM-affiliated proteins (120/531, 22.6% of total), 98 for ECM regulators (98/531, 18.4% of total) and 196 for secreted factors (196/531, 37% of total) (Figure S4). The breakdown of interactions by tumor subtypes shows large tumor-specific variations, likely reflecting the different usage of matrisome categories (Figure 1c). Additionally, a generally high correlation between tumor subtypes belonging to the same tumor and to tumors from the same tissue- and system-of-origin can be observed (Figure S5), in line with previous reports, both general and matrisome-specific [22,29]. 

**Figure 2:**
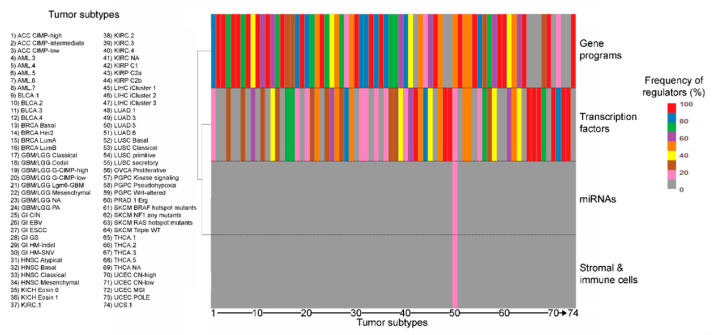
Distribution (%) of different classes of regulators (gene programs, transcription factors, miRNAs and stromal/immune content) for the landmark matrisome genes in the different tumor subtypes.

The most frequent type of explanatory variable is gene programs and pathways, which collectively dominate over the number of interactions imputed to single transcription factors, in line with their larger size, their more organic role in regulating cell activities and their wide co-occurrence in different combinations within cells from the same tumor [30] (Figure S6). Expectedly, the engagement of the different regulatory elements varies widely by tumor type and subtype, though neural signaling associates with matrisome regulation in 12 out of 24 tumor types (12/24, 50% of total tumor types), squamous differentiation, cell-to-cell adhesion and retinol metabolism in 11 each (11/24, 45.8% of total, each), proliferation and DNA repair in 10 (10/24, 42% of total) and basal signaling in 9 (9/24, 37.5% of total) (Table S1 and Figure 2), suggesting common regulatory mechanisms across different tumors. 

Several interactions have been already reported in the literature and are of interest for further investigations. For example, our approach pinpoints the link between semaphorin 4D (*SEMA4D*) and both neural signaling and cell-to-cell adhesion gene programs in neurological neoplasms (Glioblastoma Multiforme, GBM, and Brain Lower Grade Glioma, LGG), as expected from current knowledge [31,32], but also evidences a link between *SEMA4D,* Runt-related transcription factor 3 (*RUNX3*) and kinase signaling in Head and Neck Squamous Cell Carcinoma (HNSC,Table S1). This is of particular interest, since *RUNX3* is a paradoxical oncogene in HNSC (while generally being a tumor suppressor) and is connected to the transforming growth factor b (TGF-b) pathway and fibrosis [33,34], thus suggesting that the activation of this regulatory interaction plays a significant role in the microenvironmental characteristics of this neoplasia. In keeping with these findings, we evidence that *SEMA4D* is also found by our analysis in Uterine Corpus Endometrial Carcinoma (UCEC), in this case regulated by Paired box gene 8 (*PAX8*, Table S1) which is a driver of uterine neoplasms and, again, associates with fibrosis [35,36].

Only three markers (3/210, 1.43% of total) were identified as regulated by miRNAs and one (1/210, 0.48% of total) as associated with the stromal fraction, again confirming the ability of our pipeline to identify strong, cancer-specific matrisome markers whose regulation depends on intrinsic cell factors.

**Figure 3:**
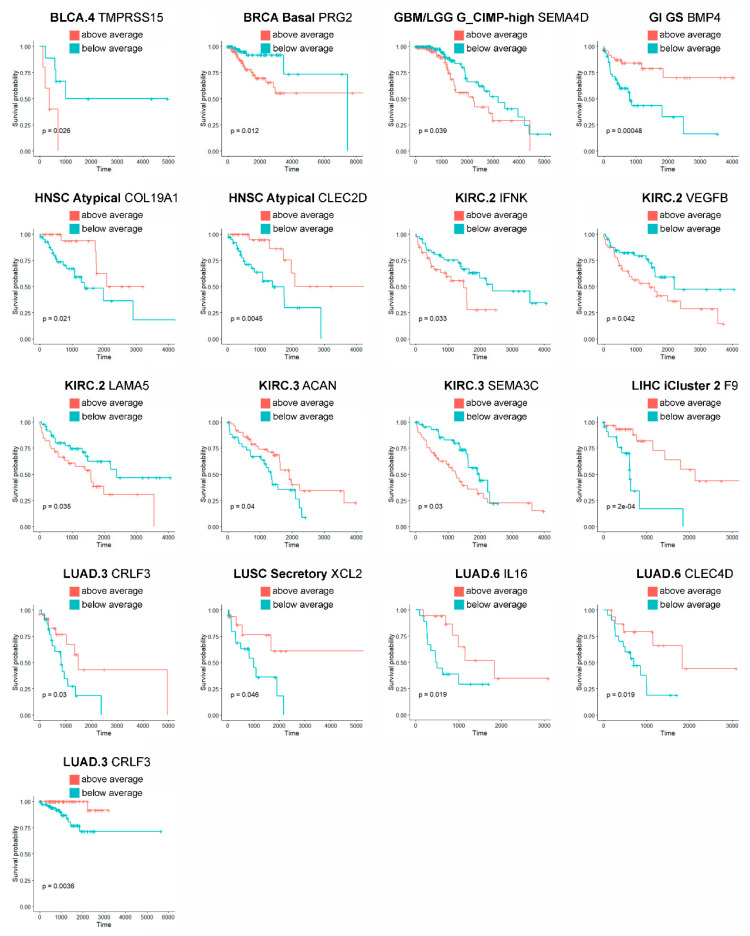
Prognostic landmark matrisome genes. Only age-independent prognostic genes (*p* value < 0.05, COX-proportional hazard model adjusted for age) are reported.

To further facilitate prioritization of our results to clinically- and pharmacologically-oriented investigations, finally, we have tested the prognostic value of the identified matrisome genes in the tumor subtypes they are marked for. In total, we pinpoint 32 prognostic genes (32/210, 15.2% of matrisome genes) in 16 tumors and 19 subtypes (66.7% and 26% of their totals, respectively), 17 of which (17/32, 53.1% of all) hold independently of patients’ age as covariate (Table S2 and Figure 3). Additionally, eight of these genes were also found prognostic by THPA analysis (Table S3) while the remaining, though not prognostic, were associated with favorable or unfavorable outcomes (data not shown).

## 3. Discussion

Understanding the breadth and the mechanisms of regulatory interactions within the TME is crucial to the development of novel therapeutic options against cancer as well as to a better understanding of the oncogenic process as a whole [37,38].

Recently, we and others have reported on the peculiar features of the tumor matrisome in terms of regulation and accumulation of mutations as well as prognostic and interventional potential [12,13,14,15]. To further the translation of these findings into pre-clinical and clinical setups, however, studies are needed to identify specific matrisome markers and targets as well as their regulatory systems in precise molecular and clinical tumor subtypes.

To this aim, we have developed a bioinformatics pipeline that enables the identification of matrisome markers in as few as 11 patients (as is the case of the LGm6 subtype of brain cancers, [39]), though the average number of patients per molecular subtype in our analysis is 81. Upon identification, all markers are then subjected to extensive modelling against almost any factor that could regulate them (like transcription factors, methylation, mutations, miRNAs, gene programs, etc.) as well as against the relative purity of the samples, to rule out possible contaminations from immune and stromal populations within the tumors [7,40]. As a result, we have identified a set of 210 tumor-specific matrisome genes which allow for a robust discrimination of precise clinical and molecular subtypes and can be fully explained by one or more regulatory features, for a total of 531 matrisome-regulator interactions by 153 unique regulators.

**Figure A1:**
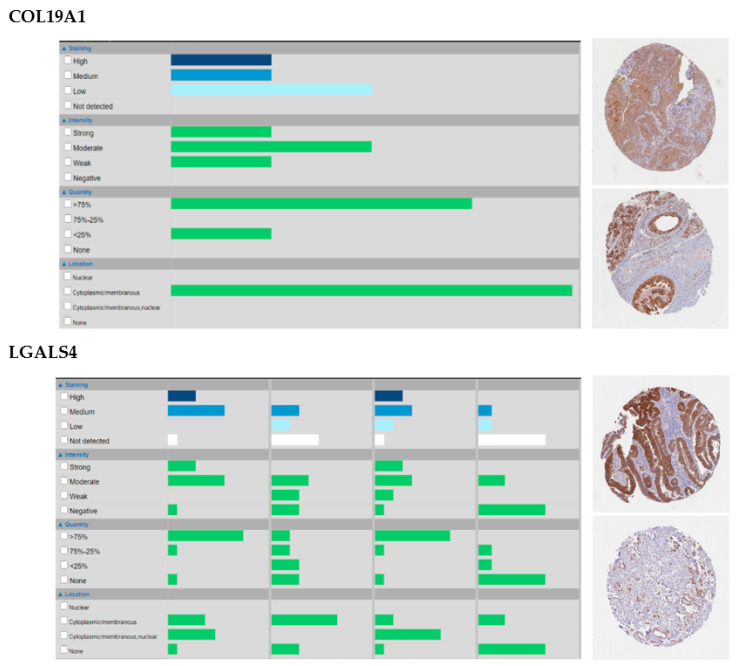
Patterns of staining, intensity, quantity and location, as well as examples, of *COL19A1* in Head and Neck Squamous Cell Carcinoma (HNSC) and *LGALS4* in STAD. Snapshots from The Human Protein Atlas (THPA, www.proteinatlas.org).

Among them, some are of particular interest because of their biological roles, prognostic value and/or their good representation at the protein level. Intersecting prognostic genes with those with high or medium protein staining in at least 50% of the samples (Table S1), for example, identifies a noticeable gene for HNSC (*COL19A1*) and one for Stomach Adenocarcinoma (STAD, *LGALS4*). In HNSC, *COL19A1* is unreported so far as a valuable target at the best of our knowledge, though it is worth noticing that it is expressed in the cytoplasm and membranes of all THPA samples and in 75% of them at a high level (Appendix A, Figure A1). In our analysis, *COL19A1* is under the control of the RAS pathway (Table S1) and is an age-independent prognostic factor for atypical HNSC, whose lower expression associates with worse prognosis (Tables S1 and S2 and Figure 3). These findings cope well with bibliographic evidence that atypical HNSC is not dependent on the RAS pathway for tumorigenesis [41], which, in this tumor subtype could thus be rather involved in other activities, and that Collagen XIX is crucial for basement membranes’ organization [42] and its loss precedes basement membrane’s invasion in ductal breast carcinoma, possibly due to the disappearance of its anti-tumoral non-collagenous domain 1 (NC1) from the TME [43,44]. Conversely, the expression of Galectin 4 (*LGALS4*), that in our analysis is an age-dependent predictor of better survival in chromosomally-unstable gastrointestinal neoplasms (GI.CIN) (Table S1) with a significant cytoplasmic and plasma membrane staining in 90% of STAD THPA samples and often at a high level (Table S2 and Appendix A, Figure A1), is reported by the same THPA as enriched in neoplasms of the gastrointestinal tract and associated organs and already considered a protective factor against gastrointestinal neoplasms [45,46]. In our results, *LGALS4* is under the control of cell-to-cell adhesion and retinoid acid metabolism, again in line with its role in stabilizing apical junctions [47] and its likely regulation by the retinoid-homologue transcription factor HNF4G [48]. Additionally, we identify only 1 marker not being regulated by an intrinsic factor (*BMP3* in lung adenocarcinoma, LUAD, subtype LUAD.5, regressing with the stromal/immune fraction) and three being regulated by miRNAs (*PLAU* in bladder cancer, BLCA, subtype BLCA.3, *TGFB2* in brain cancers, subtype GBM_LGG.Mesenchymal-like and *MMP24* again in LUAD.5), suggesting that the landmark matrisome genes we identified are of pure tumor origin and, for the very most, under the direct control of genetic programs rather than epigenetic mechanisms. Interestingly, several matrisome genes which did not make it to the final “landmark” stage were identified by sPCR and RFR as being controlled by miRNAs, confirming that epigenetic factors play a likely important role in regulating the tumor matrisome [49] though they might not concur significantly to the establishment of the “core set” of tumor-specific matrisome genes, which is likely a small but fundamental set within the TME [14].

Considering that our results span 74 tumor subtypes, there are 3.83 matrisome genes or 7.1 interactions per tumor on average. In reality, the number of matrisome genes and regulatory interactions varies significantly, with 1 to 13 matrisome genes and 1 to 22 interactions identified per tumor subtype. The ability of our pipeline to identify at least one gene or interaction in the majority of tumor subtypes that were investigated depends on a combination of backwards data modelling (from clusters to tumor subtypes) and of linear (Sparse Principal Component Regression, sPCR) and non-linear (Random Forest Regression, RFR) algorithms [50,51], which maximize the yield of matrisome variables and their regulators before the results are tested for stringent sensitivity in a series of logistic Pan-Cancer tests. This results in excellent sensitivity, which reaches an average area under the curve (AUC) of the ROC analysis of 0.87 (0.80 to 0.99, minimum to maximum). Additionally, the results we present here can be translated with good confidence into the respective proteins in independent data, provide robust tumor markers and prognostic genes and, most importantly, offer solid biological explanations to the expression of these landmark matrisome genes. 

With the growing interest in understanding the role that the matrisome plays in cancer chemoresistance and sensitivity [13,16,52,53] and the concurrent paucity of drugs targeting it [13], we believe that fine mapping of the gene regulatory networks governing the tumor matrisome will open novel therapeutic possibilities in the future and provide new tools to understand tumorigenic mechanisms and, finally, beat cancer.

## 4. Materials and Methods 

The R code sustaining this submission is available at https://github.com/Izzilab and https://rpubs.com/Izzilab/. The necessary data for the code are stored in a freely-accessible Zenodo repository (https://doi.org/10.5281/zenodo.4134099).

Raw gene-level normalized expression and phenotypical data were downloaded from UCSC Xena [25], apart from miRNA data from the miRTar database [20], purity information and protein staining intensity values from respective sources [21,28] and transcription factor–target interactions obtained from the “tftargets” package in R (https://github.com/slowkow/tftargets). Accurate data descriptors are provided within the code. Data with valid clinical and molecular subtype information (7734, the starting point for building our database) were further pre-processed for consistency, eliminating samples with missing clinical information, silent mutations and miRNAs annotated as weak entries. We calculated a stromal abundance measure as 1-CPE statistics (from [21]) and averaged gene methylation through all probes in a gene. Only the samples that passed all criteria for biological and clinical selections reported above were further processed. An R object containing all data tables, already formatted for execution, is provided in the same repository, while the raw data are available upon request to the corresponding author.

For the clustering of tumor samples based on matrisome gene expression, t-distributed Stochastic Neighbor Embedding (t-SNE) and Spearman correlation are initially used (via the “Rtsne” and the “stats” packages in R) and the results confirmed by unsupervised clustering of Gaussian Mixture Models and Uniform Manifold Approximation and Projection (UMAP)-augmented clustering (via the “umap”, “mclust” and “dbscan” packages). Optimization of t-SNE followed the steps suggested by Kobak et al. [54]. This procedure results in five matrisome meta-clusters covering all the 96 tumor subtypes for which gene expression data are available and separating blood, liver, neuroendocrine, squamous and adenomatous/sarcomatous tumors, in line with our previous report [13].

For the identification of “landmark” matrisome genes, we used a combination of cluster- and tumor subtype-level analyses. 

At the cluster-level, all samples within a tumor subtype are tested against the rest of the Pan-Cancer samples using false discovery rate (FDR)-corrected Mann–Whitney U test for each matrisome gene, after removing from the comparison those tumor subtypes that are in the same cluster as the subtype being analyzed. Removal of subtypes in the same cluster is not performed for the “adenomatous/sarcomatous” cluster (the largest) since tumor types within it show practically no correlation with each other. Next, for the same tumor subtypes, weighted gene correlation analysis (via a modified version of the standard analysis available in the “WGCNA” R package) is performed. The results from these two analyses are compared and 10X cross-validated logistic adaptive LASSO regression (tumor subtype vs. rest of the Pan-Cancer cohort, no subtypes excluded) via the “glmnet” R package is built upon common genes to filter out the non-informative ones.

At the tumor subtype-level, each matrisome gene (all matrisome genes are distributed in different parametrizations of normal distributions, checked via the “fitdistrplus” R package) in each tumor type is fitted into bimodal distribution via expectation maximization (EM) of mixtures of univariate normal (R package “mixtools”), genes are selected and allocated to each tumor type if their expression is equal or bigger than the mean + 2 times the standard deviation (2SD) of the total cohort.

The genes from the cluster- and tumor-level are finally compared and those common to both approaches are brought further to regulatory interaction modelling. These steps identify 316 (316/1027, 30.8%) unique differentially-expressed genes (DEGs). 

For the modelling of regulatory interactions, we further select subtypes with at least 10 patients and matrisome genes with at least 10 non-zero expression values. Modelling is performed via sparse principal component regression (sPCR, using the “spcr” R package) and random forest regression (RFR, using the “randomForest”, “caret” and “e1071” R packages) independently. Each regression in RFR is 10X crossvalidated. Due to their severe computational cost, both sPCR and RFR steps are performed in a University of Oulu server with 32 Xeon® cores running Microsoft R Open 3.5.3 with MKL BLAS. sPCR finishes with results for 77 tumor subtypes (77/96, 80.2% of total), explaining 259 landmark matrisome genes (259/316, 82% of landmark genes) by 374 regressors (374/109,863 potential regulatory elements, 0.34% of total). RFR finishes with results for 83 tumor subtypes (83/96, 86.4% of total), explaining 315 landmark matrisome genes (315/316, 99.7% of landmark genes) by 377 regressors (377/109,863 potential regulatory elements, 0.34% of total). Since sPCR and RFR are fundamentally different in the type of regulator-target they are best at explaining [50,51], the results at this step include 116 tumor subtype- matrisome gene-explanatory variable triplets shared by the two approaches, 1318 triplets from sPCR only and 1894 triplets from RFR only.

Interactions from sPCR and RFR are finally stacked together and each trained in logistic regression (each given interaction in the given subtype vs. the same interaction in rest of the Pan-Cancer cohort) and then tested over the same dataset. Only interactions with an area under the curve (AUC) of the ROC analysis (via the “ROCR” R package) at least 0.8 are kept. This final step produces the results presented in the text, with 210 tumor-specific matrisome genes (210/316, 66.4% of landmark genes), 153 regulators (153/109,863 potential regulatory elements, 0.14% of total) and 531 matrisome-regulator interactions in 74 tumor subtypes (74/96, 77.1% of total).

For prognostic analyses, patients in each tumor subtype (from the list above) are recursively stratified according to the expression of each matrisome gene (from the list above, above or below mean expression in the subtype). Univariate (Log-rank test) and multivariate survival analyses with age (Cox proportional hazard model) are performed with the “survival” R package. 

All graphs are drawn using the “ggplot2” R package, except for heatmaps by the “pheatmap” R package, circular correlation plot by the “circlize” R package and Kaplan–Meier curves by the “survminer” R package.

## Supplementary Materials

Supplementary materials can be found at https://www.mdpi.com/1422-0067/21/22/8837/s1.

## Abbreviations

WGCNA	Weighted gene coexpression analysis
LASSO	Least absolute shrinkage and selection operator
miRNA	Micro-RNAs
sPCR	Sparse principal component regression
RFR	Random forest regression
TCGA	The Cancer Genome Atlas
THPA	The Human Protein Atlas
TME	Tumor microenvironment
ECM	Extracellular matrix
CNA	Copy number alterations
GMM	Gaussian mixture model
EM	Expectation maximization
SD	Standard deviation
