# Supplementary material for: Machine Learning Identifies Robust Matrisome Markers and Regulatory Mechanisms in Cancer
Source: Int J Mol Sci. 2020 Nov 22;21(22):8837. doi: 10.3390/ijms21228837 (PMC7700160; doi:10.3390/ijms21228837)
Supplement: Supplementary file 1 [file ijms-21-08837-s001.zip › supps/ijms-996826_Supplementary Figures.pdf]

Type of the Paper (Communication)

Machine learning identifies robust matrisome markers and regulatory mechanisms in cancer.

Anni Kääriäinen [1,+], Vilma Pesola [1,+], Annalena Dittmann [1], Juho Kontio [1], Jarkko Koivunen [1], Taina Pihlajaniemi [1] and Valerio Izzi [1,2,3,*]

1. Faculty of Biochemistry and Molecular Medicine, University of Oulu, Oulu, Finland; anni.kaariainen@oulu.fi, vilma.pesola@oulu.fi, annalena.dittmann@oulu.fi, juho.kontio@oulu.fi, jarkko.koivunen@oulu.fi, taina.pihlajaniemi@oulu.fi, valerio.izzi@oulu.fi
2. Faculty of Medicine, University of Oulu, Oulu, Finland; valerio.izzi@oulu.fi
3. Finnish Cancer Institute; Helsinki, Finland; valerio.izzi@oulu.fi

* Correspondence: valerio.izzi@oulu.fi; Tel.: +358-46-555-3793 (V.I.)

\+ Equally-contributing authors

Supplementary Figures

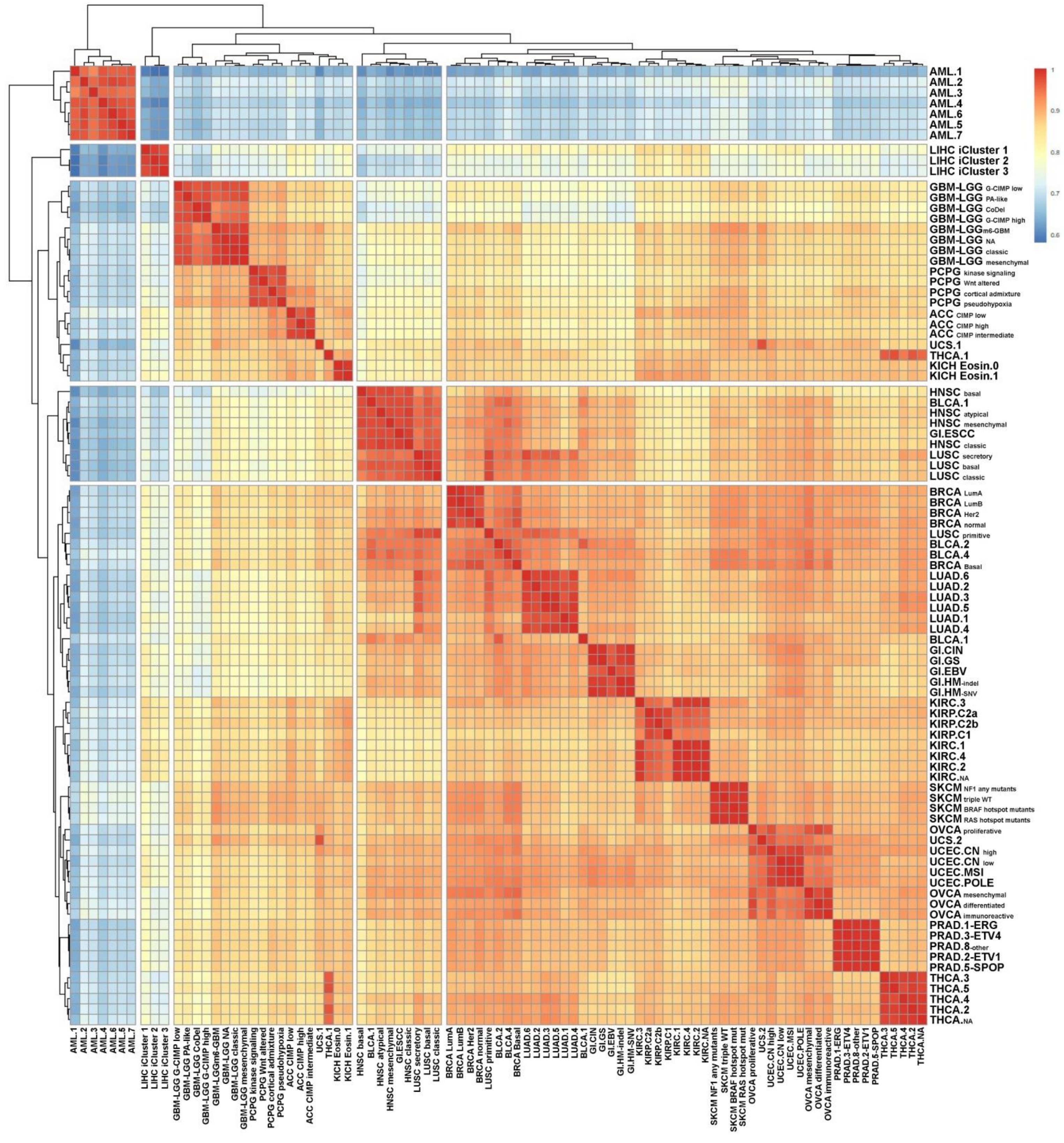

Supplementary Figure 1. Cluster structure of matrisome gene expression. The 5 clusters identified are present already at the raw matrisome data level and become progressively more evident when reducing data points to matrisome landmark genes only (as shown). Heatmap is based on Spearman correlation (thresholded at ≥ 0.5).

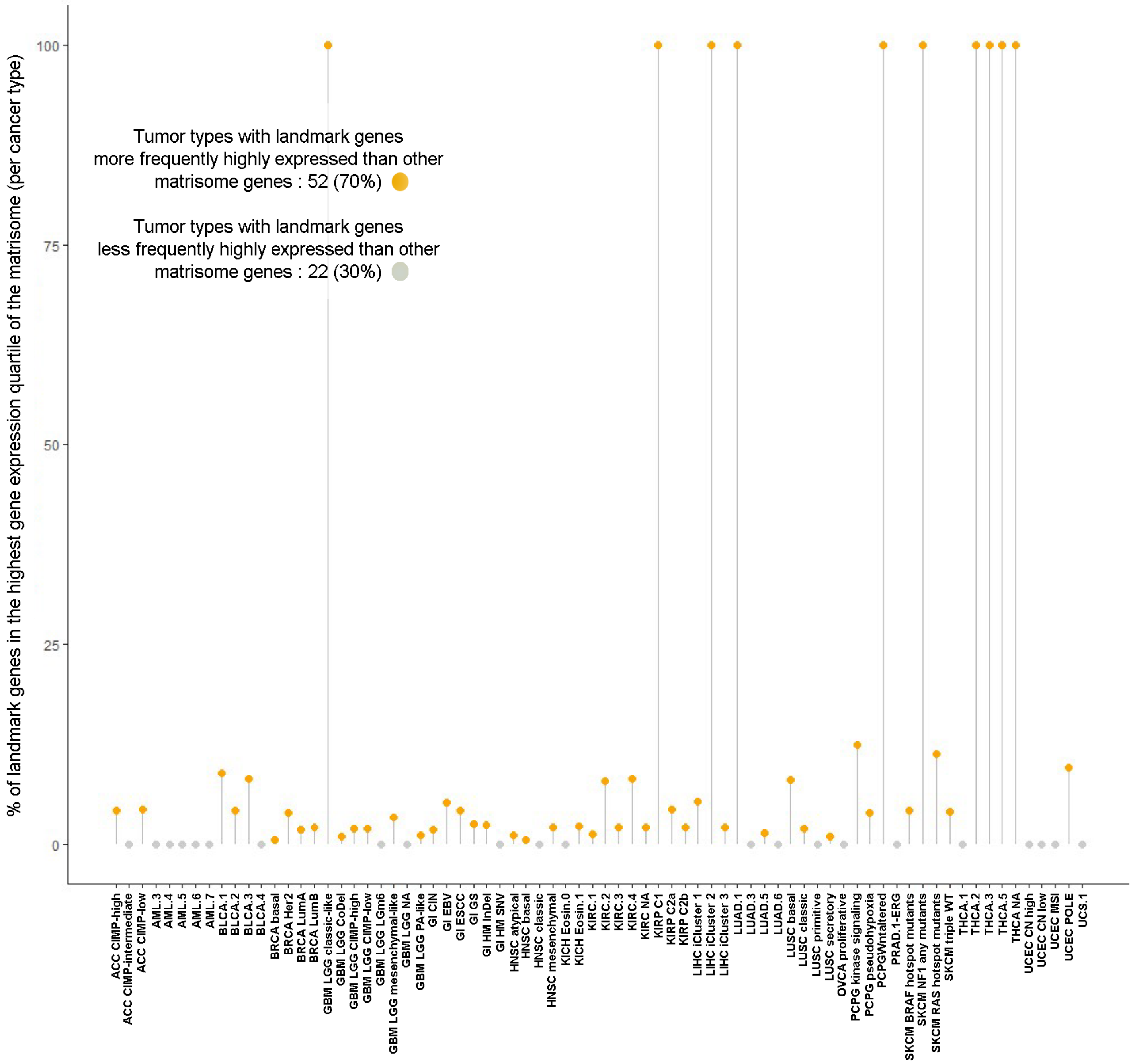

Supplementary Figure 2. Comparison (%) of times landmark genes are in the 4th quartile of matrisome gene expression *vs.* non landmark genes (per tumor subtype). Orange dots indicate a higher frequency in landmark genes, while grey (zeroed to ease interpretation) indicate a lower frequency.

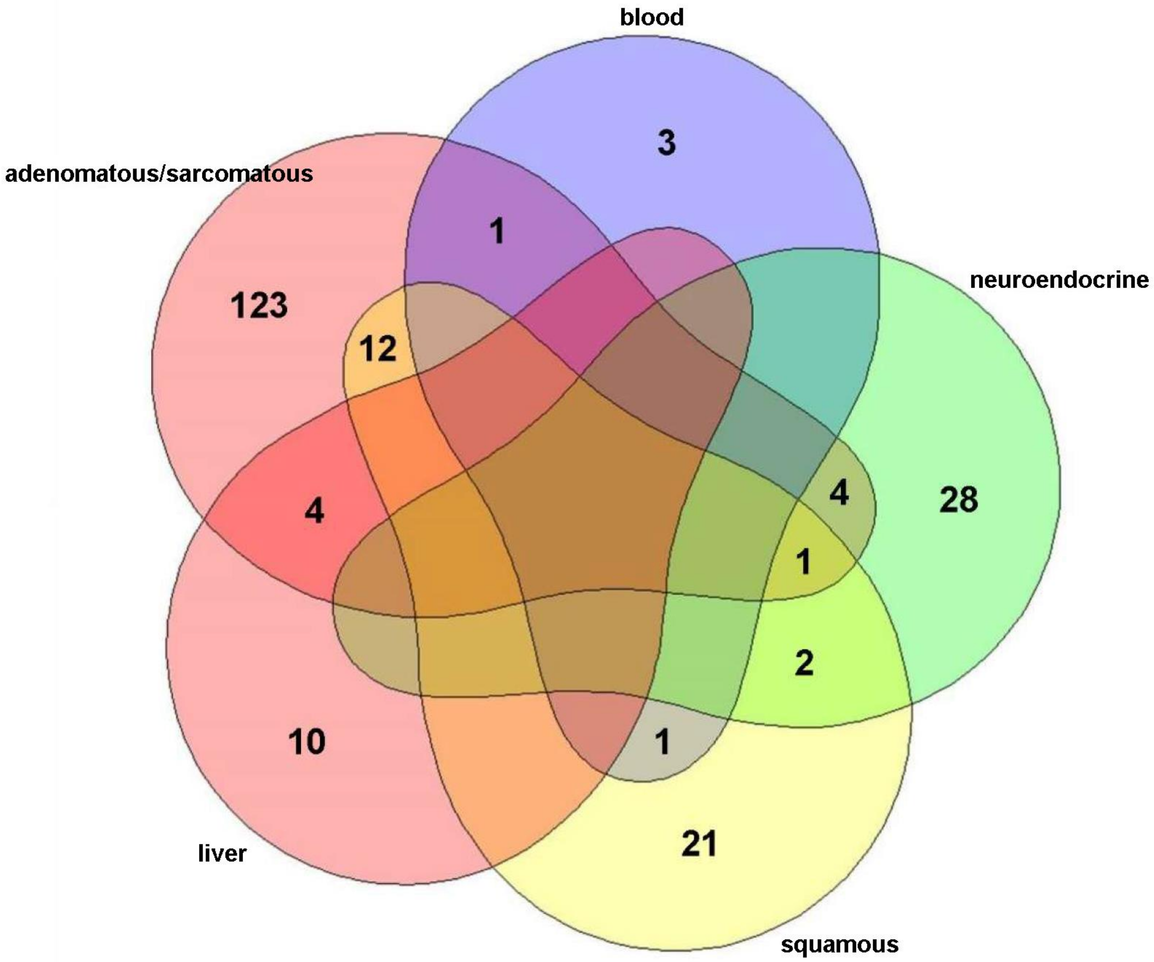

Supplementary Figure 3. Overlap in landmark genes between clusters of tumors, as previously identified and further expanded to include the neuroendocrine tumors as a cluster *per se*. Note that "blood" identifies acute myeloid leukemia landmark genes and "liver" the landmark genes for liver adenocarcinoma.

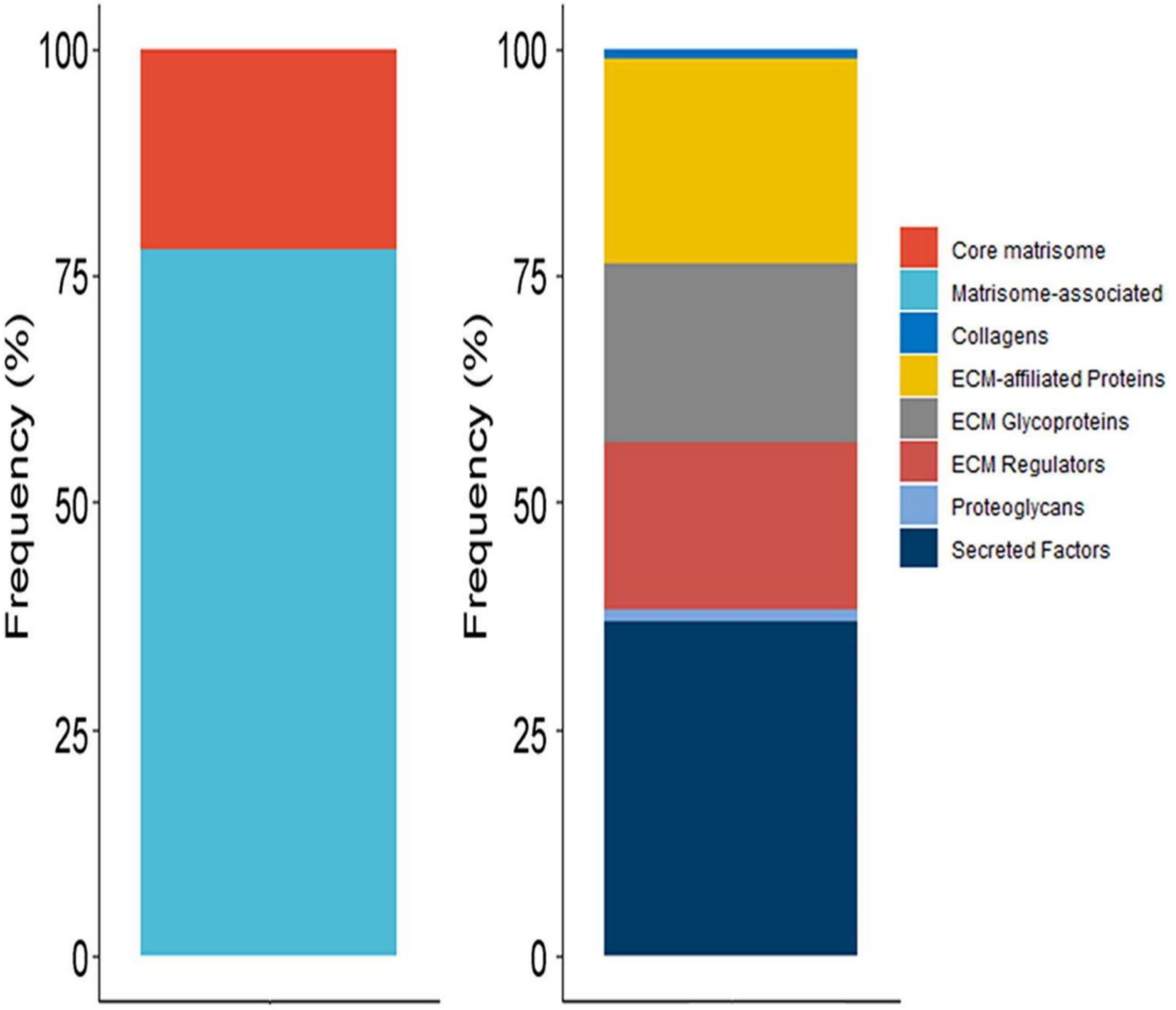

Supplementary Figure 4. % Abundance of matrisome families (core and matrisome-associated) and of matrisome categories (collagens, ECM-affiliated proteins, ECM glycoproteins, ECM regulators, proteoglycans and secreted factors) in regulatory interactions.

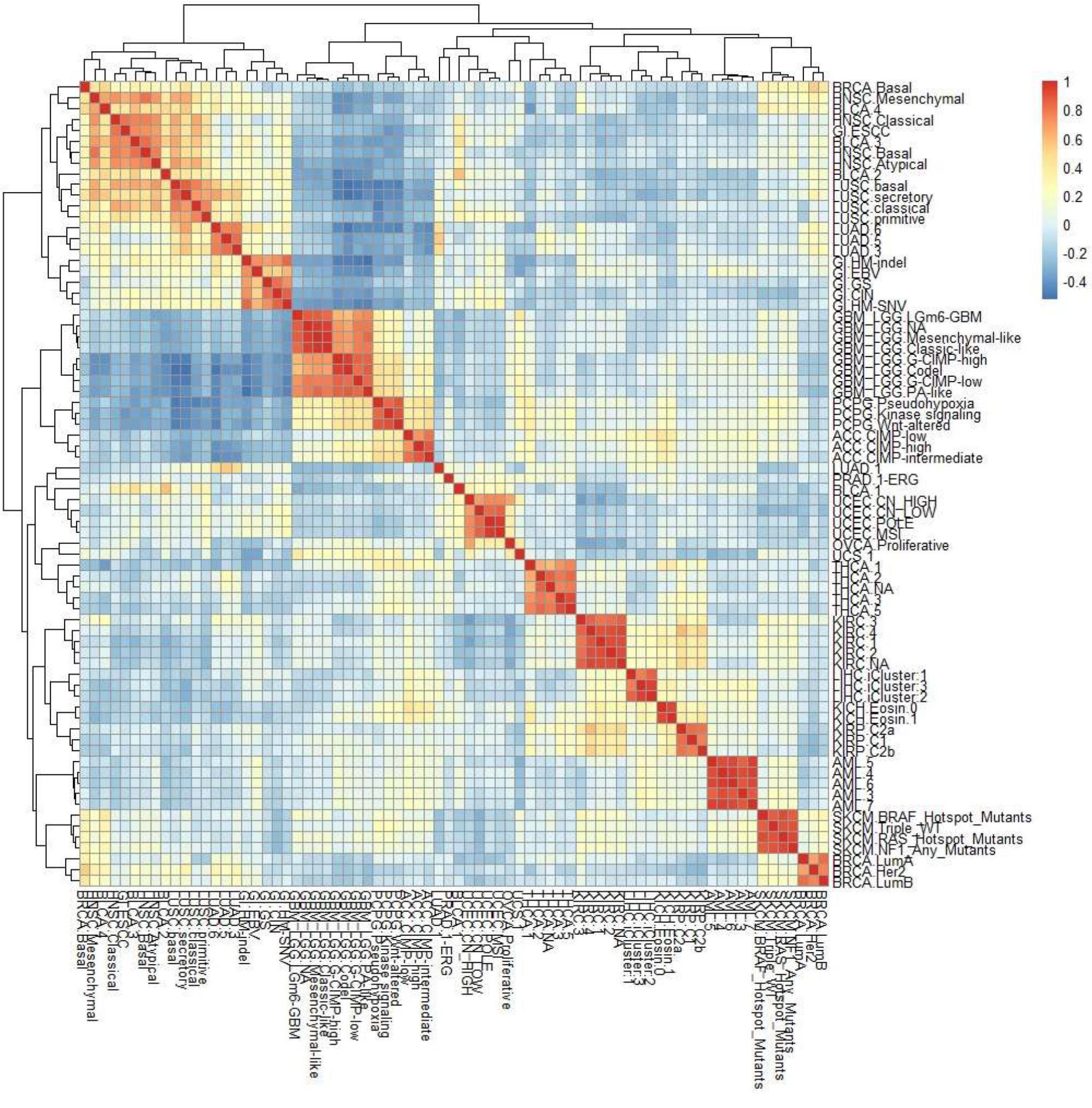

Supplementary Figure 5. Correlation (Spearman) between tumor subtypes according to matrisome landmark gene expression.

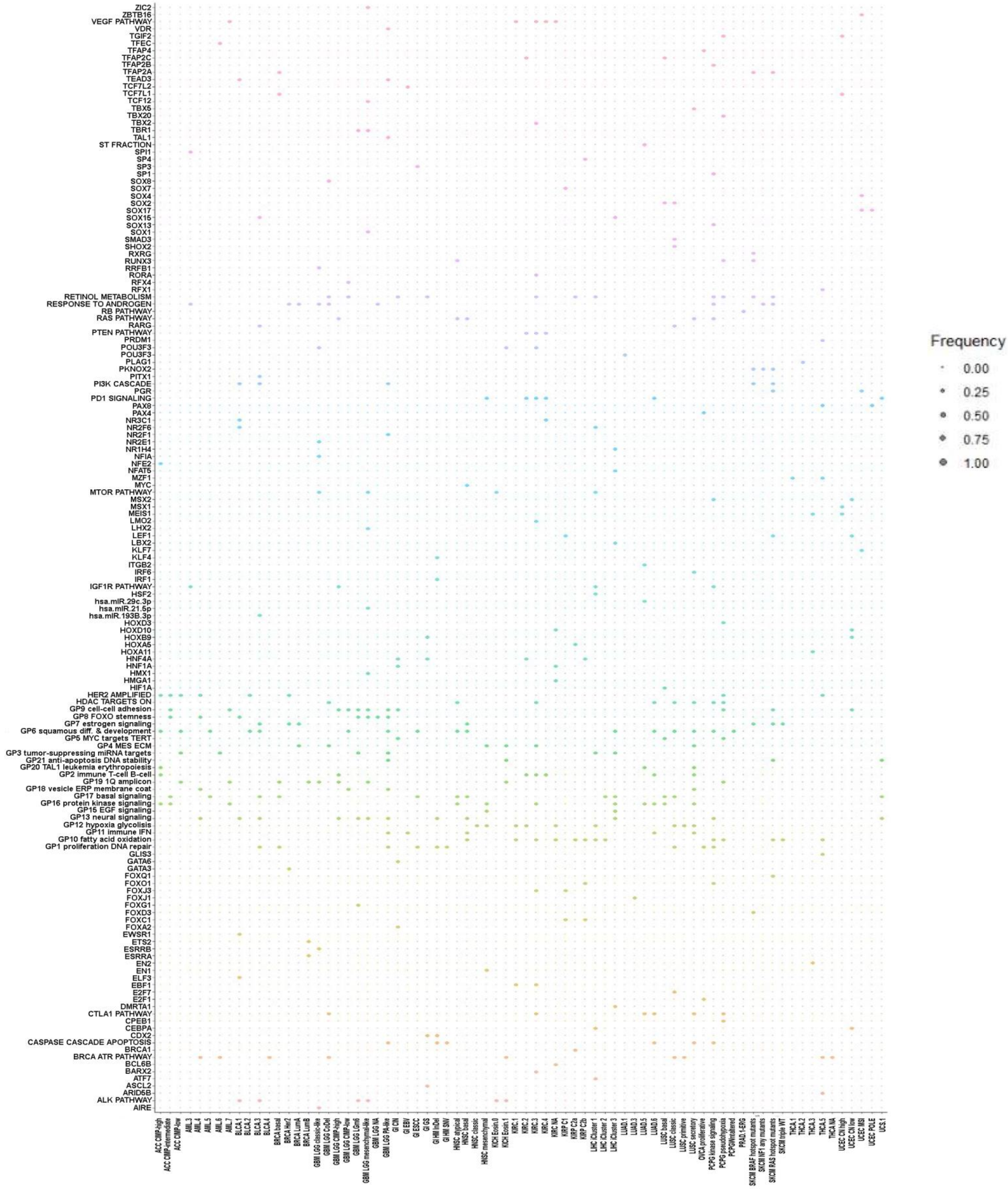

Supplementary Figure 6. Use of different regulators by the tumor subtypes. Size of the dots is proportional to the % frequency of the regulator among the total of the interactions for the given tumor subtype.
